# A population-based cohort study of mitochondrial disease and mental health conditions in Ontario, Canada

**DOI:** 10.1186/s13023-025-03688-2

**Published:** 2025-04-14

**Authors:** Laura C. Rosella, Mackenzie Hurst, Emmalin Buajitti, Thomas Samson, L. Trevor Young, Ana C. Andreazza

**Affiliations:** 1 Dalla Lana School of Public Health, University of Toronto, Toronto, Canada; 2 ICES UofT, ICES, Toronto, Canada; 3 Laboratory Medicine and Pathobiology, Temerty Faculty of Medicine, University of Toronto, Toronto, Canada; 4 Institute for Better Health, Trillium Health Partners, Mississauga, Canada; 5 Epidemiology, Biostatistics, and Occupational Health, McGill University, Montreal, Canada; 6 Department of Psychiatry, Temerty Faculty of Medicine, University of Toronto, Toronto, Canada; 7 Department of Pharmacology and Toxicology, University of Toronto, Toronto, Canada; 8 Centre for Addiction and Mental Health (CAMH), Toronto, Canada; 9 Mitochondrial Innovation Initiative, MITO2i, University of Toronto, Toronto, Canada

**Keywords:** Mitochondrial disease, Mental health, Epidemiology, Health care utilization, Health care costs

## Abstract

**Background:**

Mitochondrial disease has been linked to mental health disorder in clinical cohorts and post-mortem studies. However, a lack of population-level studies examining the relationship between mitochondrial disease and mental health has resulted in an evidence gap and creates a challenge for identifying and addressing care needs for the mitochondrial disease population. Using multiple linked population health databases in a single-payer health system that covers the full population, this study aimed to investigate the prevalence of mood disorders and other mental health conditions in patients with mitochondrial disease and to examine the joint impact of mitochondrial disease and mental health conditions on healthcare use and health system costs. To contextualize these findings, a clinical comparator cohort of multiple sclerosis (MS) patients was analyzed.

**Results:**

Overall, co-prevalent mental health conditions are common in the mitochondrial population. Double the proportion of patients in the mitochondrial disease cohort had a co-prevalent mental health illness as compared to the MS population (18% vs 9%). Healthcare utilization was highest among patients with co-prevalent mitochondrial disease and mental illness, with 49% hospitalized within 1 year prior to cohort entry (compared to 12% of MS patients with no mental health condition). Costs were likewise highest among mitochondrial disease patients with mental health conditions.

**Conclusions:**

This study presents the first comprehensive, population-wide cohort study of mitochondrial disease and co-prevalent mental health conditions. Our findings demonstrate a high burden of mental health conditions among mitochondrial disease patients, with high associated health care needs. We also find that patients with concurrent mental illness and mitochondrial disease represent a high-burden, high-cost population in a single-payer health insurance setting.

**Supplementary Information:**

The online version contains supplementary material available at 10.1186/s13023-025-03688-2.

## Background

Mitochondrial dysfunction, caused by mutations in nuclear or mitochondrial DNA, can result in a group of disorders known as mitochondrial disease [1]. It can present itself during childhood or adulthood and there are over 250 genes that have been found to be implicated in the disease [2]. Within Canada, there exists limited work on the epidemiology of mitochondrial disease derived from population data sources. Epidemiologic research has demonstrated that the prevalence of this disease is 1 in 4000, and suggests that mitochondrial disease presents a large economic burden on the healthcare system and patients [3, 4]. Additionally, mitochondrial dysfunction has been linked with mental health disorder from clinical cases [5, 6] and postmortem studies [7]. However, population-based studies examining the relationship between mitochondrial disease and mental health are lacking. Given the heterogenous nature of this disease and the missing data on the epidemiology of these conditions in Canada, it is challenging to quantify and address the care needs for those affected by mitochondrial disease and co-occurring illness.

Mitochondrial disease presents individual heterogeneity and although genetic targets exist, diagnosing this disease can be challenging [2]. Furthermore, mitochondrial disease has also been shown to present with a wide range of comorbidities, such as diabetes and Parkinson's disease [8]. This is due to the high abundance of mitochondria in almost all cells of the body. Dysfunction of the mitochondria have profound effects on neurotransmission and may contribute to changes in neuronal circuits in the brain that are associated with cognition, memory, and other forms of neuronal plasticity [9]. Thus, illnesses in which mitochondria are dysfunctional may cause disruption that has been implicated in several psychiatric conditions. For example, impaired neurotransmission is evident in patients with bipolar disorder [10], which is likely linked to specific neurodevelopmental abnormalities [11]. Because neurons depend on energy, mitochondrial dysfunction during neurodevelopment is expected to impact neurotransmission, with potentially crucial implications for the development of psychiatric conditions [12]. However, despite preliminary evidence for these associations [7, 13], few population-based epidemiological studies have examined the association between mitochondrial dysfunction and specific psychiatric conditions.

Building on previous population-based epidemiological studies [3] and using multiple population-based health databases, the objective of this study was to characterize the epidemiology of mitochondrial disease and its co-occurrence with mental health conditions in Ontario. Specifically, we sought to examine the association between mood disorders and other mental health conditions in patients with mitochondrial disease as well as examine the joint impact of mental health conditions on healthcare use and health system costs. To contextualize these findings, a clinical comparator cohort of multiple sclerosis (MS) patients was also used.

## Results

### Study population

**Fig. 1:**
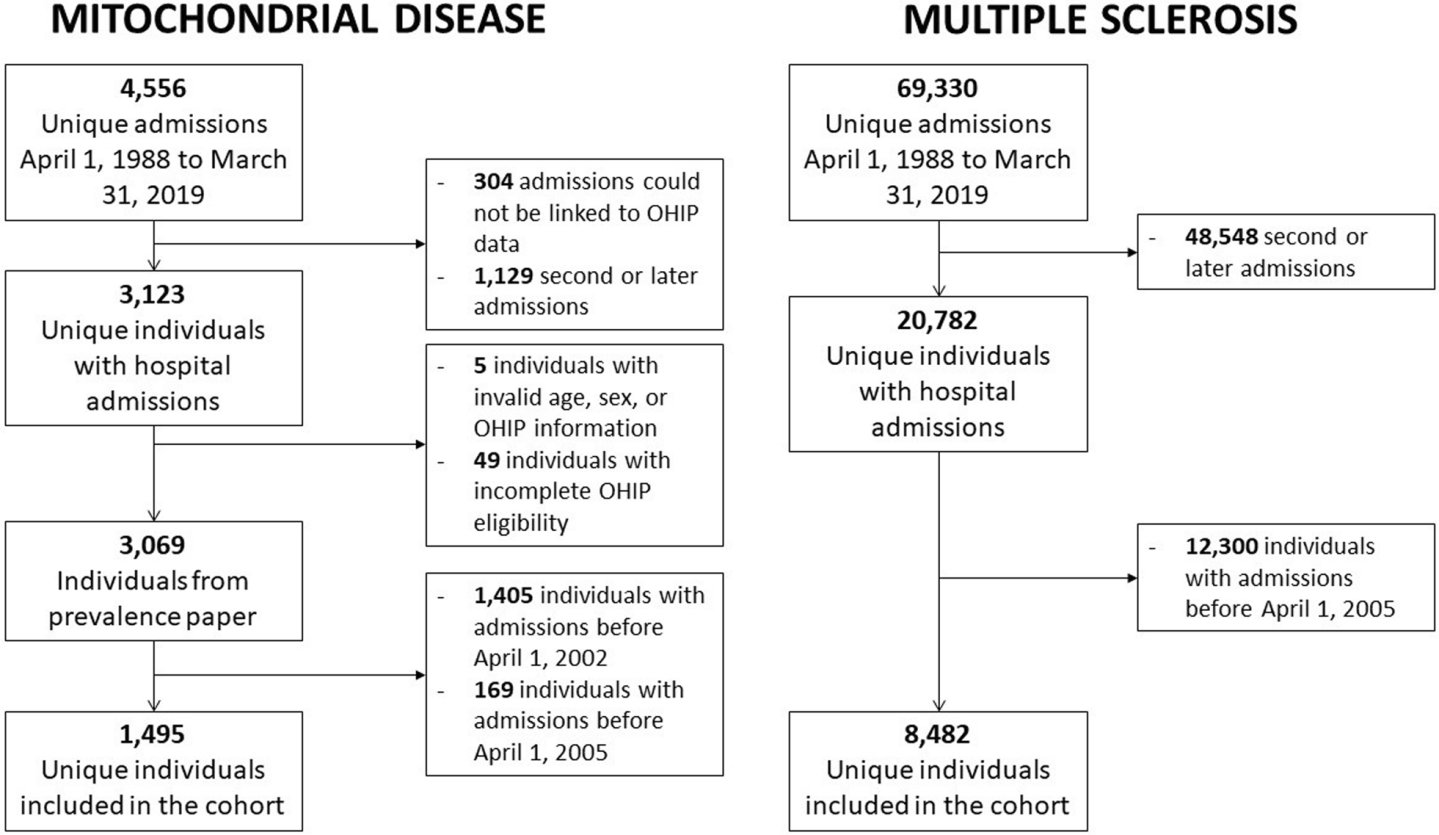
Study inclusion flow chart

A flow chart depicting the cohort creation for both mitochondrial disease and MS cohorts can be shown in Fig. 1. These datasets were linked using unique encoded identifiers and analyzed at ICES. There were 3123 unique individuals who had one or more hospitalizations with a diagnostic code indicating mitochondrial disease and could be linked to health insurance data in Ontario. There were 3069 individuals remaining after excluding incomplete Ontario Health Insurance Program (OHIP) eligibility or missing demographic information. Since a 3-year lookback window was required to capture mental health conditions and the first date to capture mental health data is April 1st, 2002, any individual with their first hospitalization indicating mitochondrial disease before April 1st, 2005 was excluded. This left a final cohort of 1495 individuals with mitochondrial disease. There were 20,782 unique individuals who had one or more hospitalizations with a diagnostic code indicating MS and could be linked to a valid RPDB record. After excluding hospitalizations from before April 1 st, 2005, a final cohort of 8482 individuals with MS was obtained.

### Cohort characteristics

Table 1 shows the demographic characteristics of the cohort, including sex, age at cohort entry (first hospitalization for mitochondrial disease or MS), and neighbourhood-level material deprivation quintile. Material deprivation refers to the inability of individuals or households to afford necessary goods and services and is a common indicator of socioeconomic status (SES) [14].

**Table 1:** Study cohort characteristics

		Mitochondrial disease patients (N = 1495)		Multiple Sclerosis patients (N = 8482)	
		N	%	N	%
Sex	M	710	47.5	2558	30.2
	F	785	52.5	5924	69.8
Age at cohort entry	0–9	290	19.4	6	0.1
	19–20	111	7.4	128	1.5
	20–29	70	4.7	620	7.3
	30–39	91	6.1	1362	16.1
	40–49	156	10.4	1675	19.7
	50–59	247	16.5	2045	24.1
	60–69	260	17.4	1576	18.6
	70–79	167	11.2	765	9
	80 +	103	6.9	305	3.6
Neighbourhood deprivation quintile	1 (least deprived)	254	17	1666	19.6
	2	257	17.2	1614	19
	3	279	18.7	1561	18.4
	4	294	19.7	1699	20
	5 (most deprived)	393	26.3	1860	21.9
Co-prevalent mental health condition	Yes	274	18.3	728	8.6
	No	1221	81.7	7754	91.4

When examining the baseline characteristics of the mitochondrial disease study population, there were no major sex differences. This is distinct from a larger proportion of the MS population was made up of females (69.8%), which was expected given the epidemiology of MS. In terms of age at cohort entry for the mitochondrial disease population, first hospitalization was most common between ages 0–9, with 19.4 percent of the study population entering the cohort before age 10. A substantial number of cohort entries also occurred in older ages, with 33.9 percent entering the cohort between ages 50 and 69. For those with MS, age at cohort entry was most common between ages 40 and 69, with 62.4% of the study population entering the cohort after age 40. Overall, a greater proportion of mitochondrial disease patients were living in lower SES neighbourhoods; 26.3 percent of those in the mitochondrial disease cohort lived in the most deprived neighbourhoods, compared to 21.9 percent of the MS cohort.

### Mental health

Overall, a much larger proportion of the mitochondrial disease cohort had a co-prevalent mental health illness as compared to the MS population, defined as one or more mental health-related hospitalization within 3 years prior to mitochondrial disease or MS diagnosis (Table 1). For those with mitochondrial disease, 18.3% (n = 274) had a co-prevalent mental illness, compared to 8.6% (n = 728) of those with MS.

Among mitochondrial disease patients with co-prevalent mental health illness, the most common diagnosis was substance-related disorders (52.2%), followed by mood/affective disorders (32.5%) and anxiety and adjustment disorders (28.5%). Among MS patients with co-prevalent mental health illness, the most common diagnoses were anxiety and adjustment disorders (44.7%), substance-related disorders (34.3%), and mood/affective disorders (27.5%). Note that these diagnostic categories are not mutually exclusive at the patient level. The prevalence of each mental health condition, by group, are in the supplement (Table S1).

### Health care utilization

Healthcare utilization for one-year periods prior to and following the index hospitalization is shown in Table 2. Healthcare utilization, both pre-and post-index hospitalization, was higher for those with one or more mental health conditions, especially for mitochondrial disease patients who also had one or more mental health conditions. For example, 48.9 percent of mitochondrial disease patients with one or more mental health conditions had at least 1 hospitalization, whereas only 39.1 percent of mitochondrial disease patients with no mental health conditions had at least 1 hospitalization. The proportion of MS patients with 1 or more hospitalizations was lower, with 12.3 percent of MS patients with no mental health conditions and 22.9 percent of MS patients with one or more mental health conditions being hospitalized at least once. This trend was similar across pre- and post- index hospitalization for other factors of health care utilization, including intensive care unit (ICU) stays and emergency department visits. However, health care utilization for outpatient care, including primary care and specialist visits, was slightly higher for MS patients. For example, MS patients had the greatest utilization of specialist visits, where 80.7 percent of MS patients with no mental health conditions and 82.0 percent of MS patients with one or more mental health conditions had 1 or more specialist visits, as compared to 73.4 percent of mitochondrial disease patients.

**Table 2:** Health care utilization prior to and after cohort entry, mitochondrial disease and multiple sclerosis patients

Health care utilization	Mitochondrial disease patients (n = 1495)		Multiple Sclerosis patients (n = 8482)	
	No Mental Health Conditions	≥ 1 Mental health condition	No Mental Health Conditions	≥ 1 Mental health condition
**1 year prior to index date**				
*Hospitalization episodes of care*				
Total hospitalizations, n	1061	368	1407	283
≥ 1 hospitalization, n (%)	478 (39.1)	134 (48.9)	956 (12.3)	167 (22.9)
Episodes of care per person, median (IQR)	1 (1–3)	2 (1–4)	1 (1–1)	1 (1–2)
Length of stay, median in days (IQR)	5 (3–10)	5 (3–9)	5 (3–9)	6 (3–10)
*ICU visits*				
Total visits, n	97	42	158	26
≥ 1 ICU admission, n (%)	77 (6.3)	31 (11.3)	124 (1.6)	24 (3.3)
Visits per person, median (IQR)	1 (1–1)	1 (1–2)	1 (1–1)	1 (1–1)
Length of stay, median in hours (IQR)	9 (5–17)	5.5 (4–12)	7 (4–13)	7.5 (4–15)
*Emergency department visits*				
Total visits, n	2572	1470	9886	2451
≥ 1 visit, n (%)	845 (69.2)	238 (86.9)	4592 (59.2)	593 (81.5)
Visits per person, median (IQR)	2 (1–4)	3 (2–7)	1 (1–3)	3 (2–5)
*Primary care visits*				
Total visits, n	8485	2101	47,453	6450
≥ 1 visit, n (%)	1096 (89.8)	241 (88.0)	6976 (90.0)	666 (91.5)
Visits per person, median (IQR)	6 (3–10)	6 (3–12)	5 (3–8)	7 (3–12)
*Specialist visits*				
Total visits, n	6347	1373	39,036	4055
≥ 1 visit, n (%)	896 (73.4)	201 (73.4)	6255 (80.7)	597 (82.0)
Visits per person, median (IQR)	5 (2–10)	4 (2–8)	4 (2–8)	4 (2–9)
**1 year following index date**				
*Hospitalization episodes of care*				
Total hospitalizations, n	1189	439	3642	545
≥ 1 hospitalization, n (%)	504 (41.3)	114 (52.6)	2157 (27.8)	275 (37.9)
Episodes of care per person, median (IQR)﻿	2 (1–3)	2 (1–3.5)	1 (1–2)	1 (1–2)
Length of stay, median in days (IQR)	5 (3–11)	5 (3–10)	6 (3–12)	6 (4–11)
*ICU visits*				
Total visits, n	135	62	435	57
≥ 1 ICU admission, n (%)	113 (9.3)	47 (17.2)	332 (4.3)	45 (6.2)
Visits per person, median (IQR)	1 (1–1)	1 (1–1)	1 (1–1)	1 (1–1)
Length of stay, median in hours (IQR)	10 (5–20)	9 (3–6)	10 (5–20)	8 (5–15)
*Emergency department visits*				
Total visits, n	2572	1470	9886	2451
≥ 1 visit, n (%)	845 (69.2)	238 (86.9)	4592 (59.2)	593 (81.5)
Visits per person, median (IQR)	2 (1–4)	3 (2–7)		3 (2–5)
*Primary care visits*				
Total visits, n	9299	1963	55,351	6495
≥ 1 visit, n (%)	1085 (88.9)	229 (83.6)	6936 (89.5)	667 (91.6)
Visits per person, median (IQR)	6 (3–11)	6 (3–11)	5 (3–9)	7 (3–12)
*Specialist visits*				
Total visits, no	6752	1376	43,416	4949
≥ 1 visit, n (%)	952 (78.0)	213 (77.7)	6889 (88.8)	678 (93.1)
Visits per person, median (IQR)	5 (2–10)	5 (2–8)	5 (3–8)	5 (3–10)

### Health care costs

Healthcare costs incurred by members of the study population before and after their index hospitalization are shown in Table 3. Overall, healthcare costs incurred by those with mitochondrial disease and MS were higher for those with concurrent mental health conditions. For example, the median cost incurred in the 12 months prior to hospitalization for mitochondrial disease patients with no mental health condition was $6859, and the median cost incurred in the 12 months post-discharge for this cohort was $21,257. Conversely, the same costs pre- and post-index date for mitochondrial disease patients with  1 or more co-prevalent mental health conditions were $10,132 and $33,363, respectively. The pre- and post-index median costs of health care for MS patients without any mental health conditions were $3984.50 and $7754, respectively, whereas these same costs for MS patients with one or more mental health conditions were $7178 and $27,314.5. Thus, costs incurred by this study population were high in both cohorts and  particularly high in mitochondrial disease patients, particularly those  with one or more mental health conditions. This trend was consistent across other categories of health care expenditure incurred by these populations including inpatient hospitalization, emergency department, and physician billing costs, where mitochondrial disease patients with one or more health conditions incurred the highest health care costs within this study population.

**Table 3:** Health care costs prior to and following cohort entry, mitochondrial disease and multiple sclerosis patients

Health care costs	Mitochondrial disease patients (n = 1495)		Multiple sclerosis patients (n = 8482)	
	No mental health conditions	≥ 1 Mental health condition	No mental health conditions	≥ 1 Mental health condition
*1 year prior to index date*				
Any health care cost, n (%)	1202 (98.4)	272 (99.3)	7706 (99.4)	727 (99.9)
Total cost, median (IQR)	6859 (1803–22,157)	10,132 (3017–33,077)	3984.5 (1729–11,425)	7178 (2732.5–18,859)
Any inpatient hospitalization cost, n (%)	479 (39.2)	134 (48.9)	958 (12.4)	168 (23.1)
Inpatient hospitalization costs, median (IQR)	9212 (3847–23,409)	11,441.5 (5234–24,346)	4769 (3343–11,897)	7842 (4018.5–15,730.5)
Any outpatient hospital cost (clinic visits), n (%)	744 (60.9)	124 (45.3)	4064 (52.4)	331 (45.5)
Outpatient hospital clinic visit costs, median (IQR)	1246 (630–2267.5)	982.5 (328.5–1951)	681 (338–1433)	655 (327–1293)
Any emergency department costs, n (%)	851 (69.7)	238 (86.9)	4629 (59.7)	601 (82.6)
Emergency department costs, median (IQR)	811 (429–1431)	1363 (710–2698)	577 (317–933)	931 (477–1651)
Any physician billings (GP and specialist), n (%)	1193 (97.7)	267 (97.5)	7596 (98.0)	723 (99.3)
Physician billings costs, median (IQR)	1676 (602–3912)	2227 (891–5174)	1105 (539–2005)	1699 (792–3183)
*1 year following index date*				
Any health care cost, n (%)	1221 (100.0)	274 (100.0)	7754 (100.0)	728 (100.0)
Total costs, median (IQR)	21,257 (9914–61,684)	33,363 (14,820–68,131)	19,539.5 (9380–44,956)	27,314.5 (12,523.5–51,715.5)
Any inpatient hospitalization cost, n (%)	1221 (100.0)	274 (100.0)	7754 (100.0)	728 (100.0)
Inpatient hospitalization cost, median (IQR)	9709 (4354–30,374)	14,624 (5819–29,682)	6534 (4506–14,290)	8123.5 (4729–18,586.5)
Any outpatient hospital cost (clinic visits), n (%)	961 (78.7)	170 (62.0)	5763 (74.3)	477 (65.5)
Outpatient hospital clinic visit costs, median (IQR)	1250 (636–2614)	954 (343–1636)	950 (363–1621)	660 (330–1310)
Any emergency department costs, n (%)	1003 (82.2)	248 (90.5)	5778 (74.5)	646 (88.7)
Emergency department costs, median (IQR)	823 (520–1481)	1977.5 (889.5–3206.5)	723 (479–1166)	1034 (616–1832)
Any physician billings (GP and specialist), n (%)	1221 (100.0)	274 (100.0)	7754 (100.0)	728 (100.0)
Physician billings costs, median (IQR)	3639 (1852–7152)	5154.5 (2600–9078)	2865 (1799.5–4829.5)	3586 (2119.5–5942.5)
*1 year following index date (excluding index hospitalization)*				
Any health care cost, n (%)	1171 (95.9)	265 (96.7)	7606 (98.1)	725 (99.6)
Total costs, median (IQR)	9011 (2027–24,511)	20,591 (5974–51,946)	9869.5 (2743–31,530)	17,866.5 (5659–39,297.5)
Any inpatient hospitalization cost, n (%)	517 (42.3)	152 (55.5)	2220 (28.6)	290 (39.8)
Inpatient hospitalization costs, median (IQR)	13,462 (5044–33,096)	13,398.5 (7735–32,440)	9058.5 (4565–21,636.5)	10,702 (5074–20,843)
Any outpatient hospital cost (clinic visits), n (%)	837 (68.6)	175 (63.9)	5576 (77.9)	560 (76.9)
Outpatient hospital clinic visit costs, median (IQR)	1249 (636–2582)	998 (622–1872)	966 (622–1637)	982 (630 − 1933)
Any emergency department costs, n (%)	715 (58.6)	217 (79.2)	3606 (46.5)	527 (72.4)
Emergency department costs, median (IQR)	674 (332–1387)	1709 (909–3017)	552.5 (269–1040)	857 (442–1684)
Any physician billings (GP and specialist), n (%)	1154 (94.5)	265 (96.7)	7541 (97.3)	722 (99.2)
Physician billings costs, median (IQR)	1960.5 (770–4488)	3621 (1284–7207)	1527 (766–2947)	2369 (1196–4077)

## Discussion

### Key findings

Overall, we identified 1495 individuals hospitalized with mitochondrial disease and 8482 individuals hospitalized with MS in Ontario, Canada, between 2005 and 2019. The prevalence of mental health conditions was significant and higher in the mitochondrial disease cohort compared to the MS cohort. The greatest health care utilization was among mitochondrial disease patients with a co-prevalent mental health illness, with 49% hospitalized compared to 12–39% in other groups. Conversely, those with MS and no mental health conditions had the lowest healthcare utilization within the study population. In terms of healthcare costs, individuals with a co-prevalent mental health illness in the mitochondrial cohort incurred the highest healthcare costs.

Our findings suggest that the mitochondrial disease and MS populations have complex healthcare needs. For comparison, previous literature has studied health system users in Ontario between 2009 and 2011, finding a median health care cost of $333 per user, with only 5% of health system users incurring $7961 or more of health care costs per year [15]. Based on this finding, a majority of our study cohort would be considered among the highest cost users within Ontario’s health system. The findings of generally high healthcare costs incurred by the mitochondrial disease population are also consistent with findings from a study done with the mitochondrial disease population in the United States [16]. This paper found that this demographic incurred over $113 million of healthcare expenditures. This expenditure went towards the direct medical costs associated with treating those with mitochondrial disease.

Healthcare utilization and costs were greatest among those with co-prevalent mental illness. Healthcare costs and utilization were higher following mitochondrial disease-related hospitalization. Since the hospitalization from that main visit was excluded from these calculations, this increase cannot be attributed to the cost of hospitalization itself. However, it can be related to post-hospitalization care, such as rehabilitation or the cost of a specialist follow-up. Our findings are consistent with other studies about health costs associated with mental illness, which suggest that patients with high mental health costs incur over 30% more costs than other high-cost patients [17]. To our knowledge, no previous study has investigated the health burden and cost associated with mental illness in a mitochondrial disease or MS patient population; our findings demonstrate that the burdens associated with these chronic conditions are exacerbated by co-prevalent mental illness.

### Limitations

The cohort used in this population-based study is restricted to hospitalized cases of mitochondrial disease because of diagnostic coding limitations in Ontario's administrative health data. Outpatient physician billings in Ontario (OHIP data) are based on a modified ICD-9 coding system and do not capture enough detail to identify rare diseases, including mitochondrial disease accurately. Patients with less acute symptoms may receive a diagnosis in Ontario (e.g. from an outpatient clinic) but would not be captured by our disease cohort. We thus consider our study population to represent a high-acuity patient population; for this reason, we have chosen a high-specificity, low-sensitivity algorithm for identifying our comparator MS cases.

Another limitation of this approach is that the date of cohort entry is based on the first hospitalization and may not reflect when a patient first becomes symptomatic or aware of their diagnosis. For this reason, pre- and post-index date periods used for assessing healthcare utilization and costs do not necessarily correspond to pre- and post-diagnostic windows. Similarly, we cannot definitively assess the onset timing of mental health conditions compared to mitochondrial disease or MS.

As with any clinical cohort comparison, the MS population is not a perfect comparator for the mitochondrial disease population, as demonstrated by the differences between these cohorts (Table 1). MS was selected as it represents a fairly common (i.e. high sample-size) neuromuscular condition with high health care utilization and demonstrated associations with mental health conditions, including in juvenile populations [18, 19]. A validated algorithm was available for identifying high-acuity MS cases, and we felt it was an appropriate comparator given its use in other studies of mitochondrial disease [20]. An amyotrophic lateral sclerosis (ALS) comparator cohort has also been used previously; however, we felt this was less appropriate given it is less common overall and in younger age groups. A healthy cohort, which is also commonly used, was not chosen as we felt it would not provide appropriate context for the health care needs of mitochondrial disease patients relative to other high-acuity clinical groups. It is possible that the results of our study could be interpreted differently had a different comparator group been used.

As the goal of this study was to describe the real-world complexity and healthcare needs of mitochondrial disease patients, our results are reported without statistical adjustment for baseline characteristics of mitochondrial disease and multiple sclerosis patient populations. It is possible that the results would differ if we had made these adjustments, given that mitochondrial patients are more commonly male (47.5% vs. 30.2%), younger (26.8% under 20 vs. 1.6%) and slightly higher deprivation (26.3% Q5 vs. 21.9%). The direction of these adjustments is unclear as these characteristics are associated differently with health care utilization (e.g. younger age typically results in less health care utilization, whereas deprivation is associated with higher utilization). Future work aiming to describe the causal relationships between mitochondrial disease and health care outcomes should consider these adjustments and others in order to draw appropriate conclusions.

## Conclusion

This study presents a comprehensive, population-wide cohort study of mitochondrial disease and co-prevalent mental health conditions in a large single-payer health system. Our findings demonstrate that the prevalence of mental health conditions is substantially elevated among mitochondrial disease patients. We also demonstrate that patients with concurrent mental illness and mitochondrial disease contribute to complexity and healthcare needs.

## Methods

### Data sources

ICES is an independent, non-profit research institute funded by an annual grant from the Ontario Ministry of Health (MOH) and the Ministry of Long-Term Care (MLTC). As a prescribed entity under Ontario’s privacy legislation, ICES is authorized to collect and use health care data for the purposes of health system analysis, evaluation and decision support. Secure access to these data is governed by policies and procedures that are approved by the Information and Privacy Commissioner of Ontario. Basic demographic information, including sex and age, was gathered from the Registered Persons Database (RPDB), which has stored data for persons registered under the OHIP at any time since 1992.

Mitochondrial disease and MS cases were identified using hospitalization records from the Discharge Abstract Database (DAD), which captures acute care hospitalizations (inpatient stays). Mental health conditions were identified through emergency department or hospital visits. Hospitalizations were captured through DAD and the Ontario Mental Health Reporting System (OMHRS). Emergency department visits were captured through the National Ambulatory Care Reporting System (NACRS) database.

Healthcare utilization was captured using data from DAD (inpatient hospitalizations and ICU stays), NACRS (emergency department visits), and physician billings from OHIP data (primary care visits and specialist visits). Healthcare costs were based on these datasets and several others, including drug payments, home care and long-term care; a full list of databases used by the costing algorithm is available in the published guidelines [21].

These datasets were linked using unique encoded identifiers and analyzed at ICES. The study period for which data were accessed was April 2005 to March 2019 for case ascertainment, with healthcare utilization and healthcare costs using data between April 2004 and March 2020.

### Cohort identification

We identified cases of mitochondrial disease as any individual hospitalized one or more times with a diagnostic code indicating mitochondrial disease in the discharge record. ICD- 10 code G71.3 was used to identify diagnoses of mitochondrial disease.

We also used a comparison cohort of patients with MS; MS has been used as a comparator climical cohort in other cohort-based studies of mitochondrial disease [12]. We likewise captured individuals hospitalized one or more times in the study period, with a diagnostic code indicating MS (ICD- 10 code G35). A validation study in Ontario found this algorithm to be low-sensitivity for general MS cases [22]. However, we believe it is appropriate for our study to capture high-acuity cases only to best align with the mitochondrial disease cohort.

Cases were excluded from either cohort if they could not be linked to a valid record in RPDB or were not OHIP eligible for at least 12 months pre- and post-admission. The eligibility exclusion was to ensure that individuals were receiving most of their health care in Ontario before and after hospitalization. Sex and age were identified based on individuals'health card records at the time of first hospital admission.

In order to determine mental health conditions prior to their first hospital admission, a 3-year lookback window was used. Since the first available mental health data is from April 2002, any person who had their first hospital admission for mitochondrial disease or MS before April 2005 was removed from the cohort for this analysis.

### Mental health conditions

ICD codes indicating mental health-related care were chosen based on a framework developed by the Mental Health and Addictions Program at ICES, formally known as the Mental Health and Addictions Scorecard and Evaluation Framework (MHASEF) [23]. This framework was developed using guidelines from the Canadian Institute for Health Information for capturing specific clinical mental health conditions from emergency department or hospital visits [24], and includes minor modifications based on Ontario-specific coding practices. MHASEF includes ICD- 10-CA codes used in DAD and NACRS, and ICD- 9-CM codes based on the 4 th edition of the Diagnostic and Statistical Manual of Mental Disorders (DSM-IV) used in OMHRS. The MHASEF was used to classify mental health care episodes by diagnosis category, into the following overarching groups: Mood/affective disorders, anxiety and adjustment disorders, schizophrenia, delusional and non-organic psychotic disorders, substance-related disorders, deliberate self-hard hospitaizations without a MHA diagnosis, and other.

### Health care utilization

Healthcare utilization was captured for all mitochondrial disease and MS patients for one year prior to and following the index hospitalization. The index hospitalization was excluded from all health care utilization measures.

A hospitalization episode was defined as the period from admission to an acute inpatient setting to final discharge from acute care, allowing for transfers between inpatient hospitals. Length of stay was based on the full episode, which may be made up of multiple individual hospital discharge records. For example, if Hospital A transfers a patient who was discharged from acute care to Hospital B, then the episode length is from admission to Hospital A to discharge from Hospital B. Emergency department visits were limited to one visit per patient per day.

Primary care and specialist physician visits were captured using physician billings and were limited to one claim per patient per physician per day. Physician specialties were based on the submitted claim and were classified as either primary care (family practice and general practice, pediatrics, and community medicine) or specialist (all other specialties).

### Health care costs

Health care costs were assessed using health administrative data from across ICES data holdings based on a costing algorithm that has been described in detail elsewhere [21]. Briefly, person-level costs are derived by combining health care utilization records with the Ministry of Health and Long-Term Care cost information. We used this algorithm to capture costs for mitochondrial disease and MS patients for one year before and after index hospitalization. As with healthcare utilization measures, costs incurred during the index hospitalization were excluded.

We measured health care costs overall and for the following specific cost categories: inpatient hospitalization, outpatient hospital clinic visits, emergency department visits, and physician billings. Costs were not standardized over time.

## Supplementary Information

Additional file 1.

## Data Availability

ICES is a prescribed entity under Ontario's Personal Health Information Protection Act (PHIPA). PHIPA authorizes ICES to collect personal health information, without consent, for the purpose of analysis or compiling statistical information with respect to the management of, evaluation or monitoring of, the allocation of resources to or planning for all or part of the health system. Legal data sharing agreements between and data providers (e.g., healthcare organizations and government) prohibit ICES from making the dataset publicly available, as the data contain sensitive and potentially identifying health information. However, access may be granted to those who meet pre-specified criteria for confidential access, available at www.ices.on.ca/DAS (email: das@ices.on.ca). The full dataset creation plan and underlying analytic code are available from the authors upon request, understanding that the computer programs may rely upon coding templates or macros that are unique to ICES and are therefore, either inaccessible or may require modification.

## Abbreviations

DAD: Discharge abstract database
ICU: Intensive care unit
MHASEF: Mental health and addictions scorecard and evaluation framework
MS: Multiple sclerosis
NACRS: National ambulatory care reporting system
OHIP: Ontario Health Insurance Program
