# Supplementary material for: A population-based cohort study of mitochondrial disease and mental health conditions in Ontario, Canada
Source: Orphanet J Rare Dis. 2025 Apr 14;20:177. doi: 10.1186/s13023-025-03688-2 (PMC11995528; doi:10.1186/s13023-025-03688-2)
Supplement: Supplementary file 1 — Additional file 1. [file 13023_2025_3688_MOESM1_ESM.docx]

**Table S1.** Prevalence of specific mental health and addictions (MHA) conditions, among mitochondrial disease and multiple sclerosis patients with any co-prevalent mental health condition.

|  | Mitochondrial disease patients (n=274) | | Multiple Sclerosis patients (N=1,507) | |
| --- | --- | --- | --- | --- |
|  | Number | Percent | Number | Percent |
| Mood/affective disorders | 89 | 32.5 | 414 | 27.5 |
| Anxiety and adjustment disorders | 78 | 28.5 | 673 | 44.7 |
| Schizophrenia, delusion and non-organic psychotic disorders | 34 | 12.4 | 105 | 7.0 |
| Substance-related disorders | 143 | 52.2 | 517 | 34.3 |
| Deliberate self-harm without MHA diagnosis | 72 | 26.3 | 199 | 13.2 |
| Other | 59 | 21.5 | 199 | 13.2 |
